# Retraction: Long Noncoding RNA-EBIC Promotes Tumor Cell Invasion by Binding to EZH2 and Repressing E-Cadherin in Cervical Cancer

**DOI:** 10.1371/journal.pone.0318789

**Published:** 2025-01-30

**Authors:** The PLOS One Editors

After this article [1] was published, concerns were raised about Fig 4 and the suitability of the reported primer sets used in the RNA immunoprecipitation (RIP) experiments.

Specifically:

- In Fig 4A, the HeLa TI10124 panel was incorrectly duplicated as the SiHa TI10124 panel.
- In Fig 4A, the SiHa TI18318 panel was incorrectly duplicated as the HeLa TI13831 panel.

The corresponding author stated that errors were made during figure preparation and provided the available original images and quantitative data underlying Figs 4A and 4B.

In editorial follow-up, PLOS contacted a member of the *PLOS One* Editorial Board regarding the methodological concerns. They stated that the primer set used to detect TI17313 in the RIP experiments was not suitable because although it detects TI17313, it was also found to amplify another genomic region containing the TMPO gene, which is expressed in HeLa and SiHa cells [2, 3]. As such, they noted that the PCR data cannot support the experimental results. Additionally, they stated that the primer sets used to detect TI10124, TI09485, TI18382, and ASK00420 may also amplify other non-targeted genomic regions.

These methodological concerns undermine the reliability of the results presented in Fig 4, and specifically, the conclusion that EZH2 binds TI17313. Although subsequent experiments showing the effect of inhibiting TI17313 are not directly impacted by the non-specific primer set used, the proposed mechanism of action via EZH2 is not fully supported. As a result, the naming of TI17313 as EZH2-binding lncRNA in cervical cancer (lncRNA-EBIC) may not be accurate.

In light of the above concerns, which undermine the reliability of the results in Fig 4 and the validity of key conclusions, the *PLOS One* Editors retract this article.

WL, FW, NXS, and CY did not agree with the retraction. QZhao, QZhang, CX, SBW, ZJJ, and SHS either did not respond directly or could not be reached. WL and NXS apologized for the issues with the published article.
